# On Structural Rearrangements during the Vitrification of Molten Copper

**DOI:** 10.3390/ma15041313

**Published:** 2022-02-10

**Authors:** Michael I. Ojovan, Dmitri V. Louzguine-Luzgin

**Affiliations:** 1 Department of Materials, South Kensington Campus, Imperial College London, Exhibition Road, London SW7 2AZ, UK; 2 Department of Radiochemistry, Faculty of Chemistry, Lomonosov Moscow State University, Leninskie Gory 1, Bd. 3, 119991 Moscow, Russia; 3 Advanced Institute for Materials Research (WPI-AIMR), Tohoku University, Sendai 980-8577, Japan; dml@wpi-aimr.tohoku.ac.jp; 4 MathAM-OIL, National Institute of Advanced Industrial Science and Technology (AIST), Sendai 980-8577, Japan

**Keywords:** amorphous copper, vitrification, glass transition, molecular dynamic simulation, Voronoi polyhedrons, congruent bond lattice, configuron, percolation, Hausdorff–Besicovitch dimensionality, set theory

## Abstract

We utilise displacement analysis of Cu-atoms between the chemical bond-centred Voronoi polyhedrons to reveal structural changes at the glass transition. We confirm that the disordered congruent bond lattice of Cu loses its rigidity above the glass transition temperature (*T_g_*) in line with Kantor–Webman theorem due to percolation via configurons (broken Cu-Cu chemical bonds). We reveal that the amorphous Cu has the *T_g_* = 794 ± 10 K at the cooling rate q = 1 × 10^13^ K/s and that the determination of *T_g_* based on analysis of first sharp diffraction minimum (FDSM) is sharper compared with classical Wendt–Abraham empirical criterion.

## 1. Introduction

Spatial distributions and displacement analysis through and between Voronoi polyhedrons are widely used to reveal structural changes in various materials in wide ranges of temperature and composition utilising molecular dynamic (MD) simulations [1,2]. The Voronoi polyhedrons are constructed using tessellations (honeycombs)-atomic centred polyhedrons which are regular or semiregular polyhedrons in crystalline materials and irregular polyhedrons in amorphous materials which are characterised by topological disorder. MD simulations are particularly effective in detecting changes that occur on vitrification of melts or melting of glasses (glass transition). Pure metals having only one sort of atoms are attractive as they enable a simplified approach compared to multiatomic compounds or metallic alloys. There are several ways in which amorphous materials including metallic systems can be produced. These include enough rapid cooling of melts, physical vapor deposition, solid-state reactions, irradiation- and pressure-induced amorphization. Amorphous Cu was produced both by extra-rapid cooling and ultrahigh pressure 14 GPa [3,4]. Many researchers utilise MD simulations to investigate its behaviour and properties [5]. The most intriguing question is about structural changes at the glass transition which was analysed using MD simulations for Fe [6,7], Ni [8,9], Cu [10], Al [11] as well as for other metals [12]. We aim here to analyse the glass transition (vitrification) of Cu revealing the structural differences below and above the glass transition temperature (*T_g_*) and utilising the pair distribution functions, mainly their first sharp diffraction minimum (FSDM) as a tool to identify the *T_g_*. 

## 2. Structural Differences between Glasses and Melts

It is believed that both glasses and liquids have almost the same topologically disordered structure despite of the fact that their properties are completely different. Variations of specific volume and thermodynamic parameters of amorphous materials such as enthalpy, entropy, heat capacity indicate on a second order-like phase transformation at *T_g_* whereas attempts to directly unveil the structural differences of atomic distributions below and above the *T_g_* fail as standard symmetries which are typically broken on phase transformations remain unchanged e.g., translation and rotation symmetries. The structural difference between glasses and melts is however unambiguously revealed by the set theory which accounts for the Hausdorff–Besicovitch dimensionality of bonding system of materials [13,14,15,16]. 

The set theory as a branch of mathematical logic that studies abstract sets which has been used to characterise the configuron phase formed in amorphous materials out of broken chemical bonds termed configurons. The configuron phase behaves differently in glasses and melts forming a condensed-like phase in melts and occurring as a point-like (defect) phase in glasses. It has therefore different Hausdorff–Besicovitch dimensions in melts and glasses. The Hausdorff–Besicovitch dimension is defined as the limit D = lim(ε→0)[logN(ε)/log(1/ε)] where N(ε) is the number of boxes of side length ε required to cover the set. It has been revealed that the set has the Hausdorff–Besicovitch dimension D when N(ε) grows proportionally to (1/ε)^D^ as ε tends to zero. The dimensionality of the set of configurons (of the configuron phase) changes at the glass transition temperature (*T_g_*) from 0 (which is typical for point-like systems such as gases) to D = 2.55 ± 0.05 (which is fractal and is typical for liquids) above it. The stepwise change of Hausdorff–Besicovitch dimension of the set of configurons is due to the formation of the condensed configuron phase above the *T_g_* and has as consequence the appearance of a kink in the first sharp diffraction minimum of scattered X-rays or neutrons [17,18]. Thus, the set theory provides clear evidence of structural differences between glasses and melts as both glassy and liquid structures near *T_g_* are disordered, however they have different Hausdorff–Besicovitch dimensions. 

## 3. Configurons in Amorphous Cu

**Figure 1:**
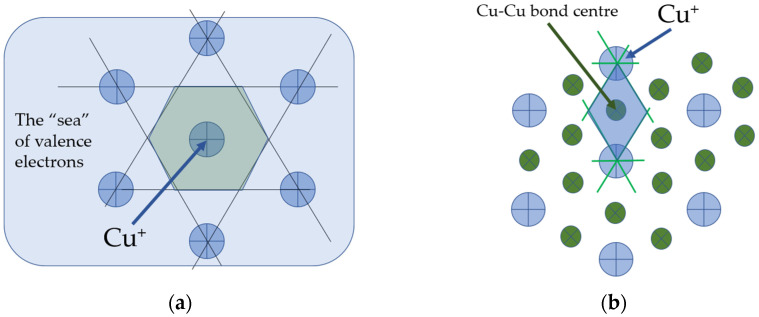
(**a**) The atomic lattice; (**b**) the corresponding congruent bond lattice (CBL) of Cu. Shadowed areas correspond to Cu-centred (**a**) and bond-centred (**b**) Voronoi polyhedrons.

A powerful way to investigate the structure of materials is to analyse the structure of chemical bonds between atoms which constitute the condensed matter either solid or liquid. The initial fully connected atoms in a solid material may lose their solid-like behaviour on breaking the chemical bonds. Kantor and Webman have proved in 1984 that the rigidity threshold of an elastic percolating network is identical to the percolation threshold [19]. This means that finding the percolation threshold as a function of temperature, we can find the critical temperature when the solid-like behaviour changes to a liquid-like that is for amorphous materials we can find the *T_g_* assigning it to the temperature when the percolation via broken bonds occurs [20]. In addition to that, once the percolation cluster made up of configurons is formed, its structure has a fractal geometry rather than 3D, its Hausdorff–Besicovitch dimension is ≈2.5 instead of 3 [13,14,15,16]. The utilisation of chemical bond concept in metals is not straightforward as for metals bonding is provided by the delocalised valence electrons from the s and p orbitals of the metal which form a common “sea” of electrons that surrounds the positively charged atomic nuclei (Figure 1a). 

We follow here the concept of configurons first developed by Angel-Rao [21] based on concepts developed for metallic glasses by Egami et al. [22] who emphasised that local atomic connectivity is changed by gaining or losing a nearest neighbour (topological excitation) and introduced local configurational excitation as the elementary excitation in high temperature liquids [23]. The congruent bond lattice (CBL) replaces the system of N strongly interacting ions by the system of N’ = N(Z/2) weakly interacting chemical bonds where Z is the coordination number (Z = 12 for metallic Cu). Figure 1a,b schematically shows both the almost regular lattice and the corresponding CBL of Cu where we accounted that the [111] plane of face-centred cubic lattice is a hexagonal grid. The Voronoi polyhedrons are constructed by first picking a lattice point, which is the Cu^+^ ion the case (a) and the centre of Cu-Cu bond in case (b) after which lines are drawn to all nearby lattice points followed by drawing perpendicular planes which in such a way enclose the smallest volume termed also as the Wigner–Seitz primitive cell. The Voronoi polyhedrons are typically approximated by spheres of equal volume characterised by a single parameter–their radii or diameters [16]. 

**Figure 2:**
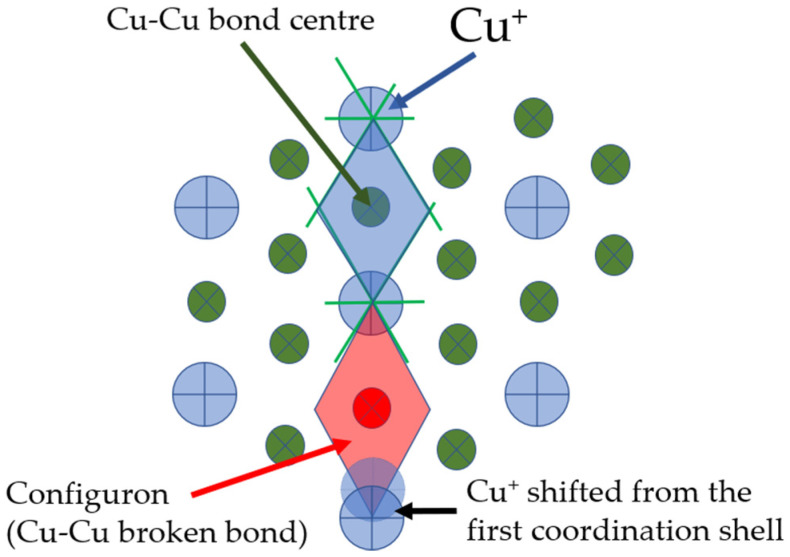
Schematic of a configuron shown by its Voronoi polyhedron shadowed in red colour in amorphous Cu. Formation of configuron is linked to the displacement of Cu^+^ from the first coordination shell [16,18] and local atomic connectivity change by losing a nearest neighbour [23].

At the nil temperature all chemical bonds are intact (unbroken), and the amorphous Cu is a solid-like material: it is a glass. On increasing the temperature some of the chemical bonds are broken forming configurons which at low temperatures are point-like entities such as point defects in solids. Figure 2 shows formation of a configuron in the CBL of amorphous Cu with a notable shifting of the unbound Cu^+^ and an increased volume of configuron Voronoi polyhedron compared to original bond Voronoi polyhedron.

In such a way we can utilise the configuron concept in metallic systems which can be described as a displacement of an atom out of the first coordination shell with the neighbouring adjustments and has as a microscopic result the shift on one or more atoms from the first coordination shell (Table 1 of [16]). The atomic displacements caused by configuron formation can be revealed analysing the scattering of X-rays or neutrons through pair distribution functions *PDF*(*r*), moreover it was shown that variations in the first sharp diffraction minimum (FSDM) of *PDF*(*r*) contain the information on structural changes in vitrifying/melting materials at the *T_g_* through the onset of the FSDM temperature variation kinks [18]. 

The following sections demonstrate the utilisation of configuron concept for amorphous copper and illustrates the methodology of identification of *T_g_* based on temperature behaviour of FDSM. The higher the temperature the more chemical bonds are broken until the critical temperature has been achieved where percolation via broken chemical bonds occurs. Because the rigidity threshold of an elastic percolating network is identical to the percolation threshold [19] the amorphous Cu transform at this temperature from the glass into a liquid, i.e., the glass transition occurs. Further atomic displacements above *T_g_* due to configuron formation will follow a different law because the material has become liquid. Indeed, the condensed phase of configurons strongly changes the behaviour of the material because a new path of facilitated motion of atoms appears. Additional availability for atomic motions is ensured and the material shifts from the solid-like to the gas-like type behaviour [24,25,26,27,28]. The Benigni’s liquid-like B phase in the 2-state model [29] is formed and the state of atoms within the percolation cluster made up of configurons is assimilated to a gas-like type with consequent contributions to the heat capacity of material and its mechanical properties [16,29]. 

## 4. Experimental

**Figure 3:**
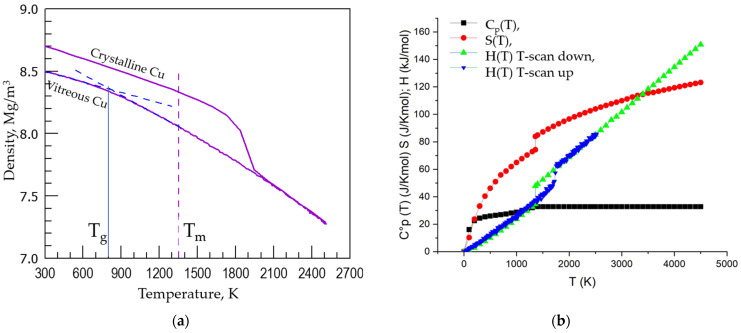
(**a**) Density changes on heating and cooling of crystalline and vitreous Cu respectively where *T_m_* is the melting temperature (1358 K) and *T_g_* is the glass transition temperature obtained at the given cooling rate q = 1 × 10^13^ K/s. (**b**) Thermodynamic functions of Cu as function of temperature demonstrating characteristic changes at *T_g_* and *T_m_*. There is no change at *T_g_* for C_p_(T) because the data presented are for crystalline Cu taken from the Reference [37] and the change is seen only at *T_m_*. Data of current work are given in the blue colour.

The vitrification of Cu was simulated on continuous cooling and isothermal annealing via classical molecular dynamic (MD) computer simulation procedure at constant pressure using the software package (LAMMPS) [30]. The simulation was performed at 1 fs time step using the embedded atom potential for Cu taken from Ref. [31] under periodic boundary conditions with stabilised temperature and pressure. The cubic cell size was about 10 nm aiming to let crystallisation to occur. A crystalline cell containing 256,000 atoms was heated to the temperature T = 2500 K at 1 × 10^14^ K/s and held for 10 ps obtaining the melt confirmed by the radial distribution function form and by time stabilisation of the materials density. Equilibrium liquid state was obtained at 2500 K in less than 1 ps. Holding liquid for longer times caused no visible changes in the liquid structure and density. To obtain vitreous Cu the melt was cooled down with the rate as high as q = 1 × 10^13^ K/s keeping the accuracy of temperature upon simulation within ±5 K. It is known that at lower cooling rates of 5 × 10^12^ K/s and lower the molten Cu started to crystallise [32]. Thermostat and barostat were used to control the temperatures and pressures [33,34,35]. The “OVITO” software package [36] was used to visualise and analyse simulation results while the adaptive common neighbour analysis was used to detect structural changes. The density of Cu as a function of temperature is shown in Figure 3a.

**Figure 4:**
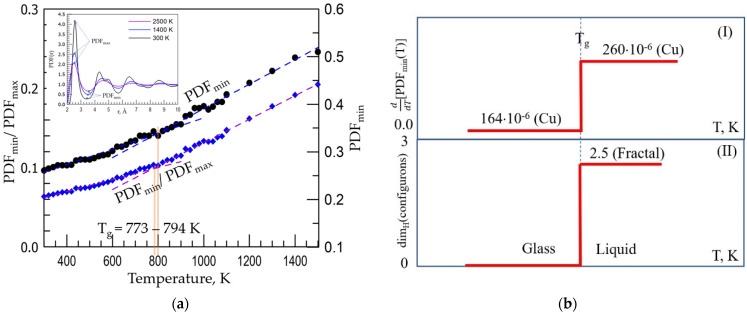
(**a**) The values of pair distribution function first minimum PDFmin of Cu following the method proposed in [18] and ratios of PDF_min_/PDF_max_ after Wendt–Abraham criterion [38] as a function of temperature where the inset shows the definitions of parameters used with PDF(r) given for three temperatures *T* = 2500, 1400, and 300 K respectively. (**b**) The prominent changes at the Cu glass transition temperature *T_g_* = 794 ± 10 K of FSDM (I) and Hausdorff–Besicovitch dimension of configurons phase dim_H_(configurons) (II).

The *T_g_* roughly estimated from the temperature dependence of density of vitreous Cu (Figure 3a) at such high cooling rate (q = 1 × 10^13^ K/s) is approximately 800 K. A more exact determination of *T_g_* is possible using either the empirical Wendt–Abraham criterion [38] which is based on the analysis of the pair distribution functions *PDF*(*r*) peak minimum to maximum ratio or using the more sensitive method proposed by authors [18]. Indeed, the structure of condensed matter (crystals, glasses, melts) can be unveiled using the *PDF*(*r*) which can be also used to understand the changes that occur in materials on cooling/heating, including the structural modifications on vitrification of melts. It was found that variations in the first sharp diffraction minimum (FSDM) of *PDF*(*r*) contains the information on structural changes in vitrifying/melting materials at the *T_g_*. A useful application from this discovery was the method proposed for determining the glass transition temperature which assigns the *T_g_* to the temperature of the onset of the FSDM variation kink [18]. Experimental data were processed using the R-statistic with segmented package: the intersection point of two fitting lines produced by segmented fitting using a computer program R, namely an environment for data analysis and graphics [39] and Akaike criterion [40] for segmented regression [41]. Figure 4 shows the results of utilisation of these criteria for amorphous Cu based on data obtained in this work. 

As seen from the inset of Figure 4a, the FDSM (*PDF_min_*) is progressively increased with the temperature change: the higher the *T*, the higher FDSM. The temperature changes of FDSM are linear with temperature while the rate of growth d*PDF_min_*/dt changes stepwise from a lower value to a higher one at the *T_g_* as observed by Wendt–Abraham who proposed a practical criterion to identify the glass transition temperatures based on data on temperature dependence of $\Phi (T)=\left(PDF_{min}/PDF_{max}\right)$ [38]. Thus, the following equations hold [18,38]:

(1) $$PDF_{min}\left(T\right)=a_{g}T+b_{g},\;\mathrm{at}\;T\;<\;T_{g}$$

(2) $$PDF_{min}\left(T\right)=a_{l}T+b_{l},\;\mathrm{at}\;T\;>\;T_{g}$$

(3) $$\Phi (T)=c_{g}T+d_{g},\;\mathrm{at}\;T\;<\;T_{g}$$

(4) $$\Phi (T)=c_{l}T+d_{l},\;\mathrm{at}\;T\;>\;T_{g}$$

The parameters of these equations are presented in the Table 1 as found from statistical processing of experimental data.

The *T_g_* based on *PDF_min_*(*T*) or Φ(T) are found accordingly from the Equations (5) and (6):

(5) $$T_{g}=(b_{g}-b_{l})/(a_{l}-a_{g}),\;$$

(6) $$T_{g}=(d_{g}-d_{l})/(c_{l}-c_{g})\;$$

The *T_g_* of Cu is hence found as high as 794 ± 10 K from the FSDM temperature dependence and 773 ± 12 K from the Wendt–Abraham criterion based on temperature dependence of $\Phi (T)=\left(PDF_{min}/PDF_{max}\right)$ at q = 1 × 10^13^ K/s. Both data are consistent with the rough estimate based on density change with temperature *T_g_* = 800 K (Figure 3a and Figure 4b reveals that the change of the growth rate of FSDM at *T_g_* given by d*PDF_min_*/dT = (a_l_−a_g_) is 93 ppm which is significantly larger compared with the change of the growth rate of the Wendt–Abraham criterion at *T_g_* given by dΦ(T)/dT= (c_l_−c_g_) = 72 ppm, therefore the identification method based on FSDM is considerably sharper compared with the Wendt–Abraham criterion which stands in line with previous data for amorphous Ni [18].

## 5. Discussion

Glass transition phenomena are observed universally. In practice, any liquid can be vitrified (transformed from liquid to a glass) if the cooling rate is enough high to avoid crystallisation. However structural changes that occur on vitrification are almost undetectable despite qualitative differences of characteristics of amorphous materials above and below *T_g_*. This makes difficult treatment of glass transition as a real phase transformation (which can be non-equilibrium). Often the glass transition is considered as just a gradual although considerable change of material viscosity with an arbitrarily defined glass transition temperature at which the equilibrium viscosity of the melt reaches 10^12^ Pa·s [42]. The definition of a glass as an amorphous material at viscosities above 10^12^ Pa·s is inconsistent for at least the reasons that (i) the viscosity is a continuous function of temperature in contrast with derivative thermodynamic parameters such as heat capacity, and (ii) the viscosity is not necessarily equal to 10^12^ Pa·s at *T_g_* [17]. Also, in many marginal glass-formers equilibrium viscosity of 10^12^ Pa·s (or any other similarly high value) can never be reached owing to competing crystallization process. Experimentally the glass transition is observed as a second order-like phase transformation. Indeed, the material volume and entropy are continuous functions of temperature exhibiting kinks at *T_g_*, and discontinuities are observed only for their derivatives. Namely these characteristics are used in practice to detect where transformation occurs, that is to detect the *T_g_*. Because of universally observed thermodynamic evidence of second order-like phase transformation in amorphous materials the term “calorimetric glass transition” was coined—see Chapter 3.2 of Ref. [43].

**Figure 5:**
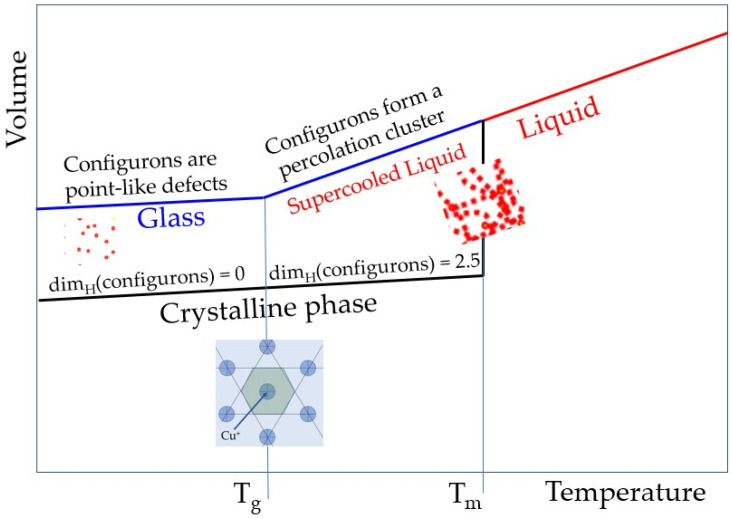
A sketch of the temperature dependence of the specific volumes of glass (*T* < *T_g_*), and both supercooled (*T_g_* < *T* < *T_m_*) and stable melts (*T* > *T_m_*). For amorphous copper we have found that *T_g_* = 794 ± 10 K at q = 1 × 10^13^ K/s, and the melting temperature is known as *T_m_* = 1358 K. The slope of lines is schematically exaggerated while the linear thermal expansion coefficient is 49.5 × 10^−6^ K^−1^ for of the Cu glassy phase and 800 × 10^−6^ K^−1^ for the Cu melt phase [46].

The crucial argument in treating the vitrification as a phase transformation of an amorphous materials at *T_g_* is related to the possibility to observe structural changes at the glass transition. Obvious symmetry changes occur at crystallisation with the formation of an ordered (most often periodic, although for quasicrystals not necessarily) anisotropic structure. The structure of glasses is, however, also disordered similarly to that of liquids (though somewhat more ordered on the medium range scale of 0.5–1 nm). This makes almost impossible to distinguish structurally a glass from a melt based on distribution of atoms. Here we show how to utilise data of neutron and/or X-ray diffraction to reveal the structural differences between liquids and glasses. The almost undetectable changes of the structure of amorphous materials at glass transition can be revealed based on the concept of broken chemical bonds (configurons). We emphasise that the configuron percolation theory (CPT) treats transformation of glasses into liquids at glass transition as an effect resulting from percolation via broken chemical bonds (configurons). It is important that MD simulations showed that low atomic density Voronoi polyhedrons percolation clusters are formed in the liquid state made whereas there are no such clusters in the solid (glassy) state [44]. The CPT envisages that structurally melts have a fractal geometry of chemical bonds with broken chemical bonds forming extended (macroscopic) fractals and because of that a liquid-like behaviour, and glasses have a 3D geometry of chemical bonds with point-type broken chemical bonds having a 0D geometry and because of that a solid-like behaviour (Chapter 3.1 in [45]). Figure 5 shows the temperature dependence of amorphous Cu volume on glass transition and melting and related changes of Hausdorff–Besicovitch dimensionalities of configuron phases dim_H_(configurons).

The interatomic chemical bonds in condensed materials can be broken by fluctuations of temperature, also by high pressure and ionizing radiation. Bond breaking processes inevitably lead to shifts of atoms from their original lattice (which is not necessarily ordered) places. In order to experimentally reveal the presence of configurons which are elementary excitations rather than material entities one can detect the atomic displacements caused by configuron formation. The displacements of atoms from lattice sites can be detected using neutron and/or X-ray diffraction. While the temperature causes bond-breaking processes and the higher the temperature, the more configurons are formed and thus more atoms are displaced out of the first coordination shell. Namely the atoms are shifted to distances corresponding to minima of PDF(r) due to configuron formation [18]. The higher the temperature, the higher is thus the *PDF_min_*. Moreover, on approaching and crossing the *T_g_*, the temperature behaviour of *PDF_min_* will change its character. The change occurs because the topological organisation of chemical bonds (the geometry of chemical bonds) in the liquid state of matter drastically changes from 3D which is characteristic to solid state to the fractal geometry which is characteristic to the liquid state. Thus, the FSDM contains information on structural changes in amorphous materials at the *T_g_*. A method for determining the *T_g_* is hence to assign it to the onset of kink of FSDM and the proposed method is more sensitive than the Wendt–Abraham criterion based on the analysis of diffraction peaks [18,47,48]. It is also notable that the Wendt–Abraham criterion supposed that at *T_g_* the equality holds $\Phi (T_{g})$ = 0.15 [38]. This however is not always an exact relationship [49] and one can see from Figure 4a that for the amorphous Cu we get $\Phi (T_{g})=0.1$, whilst using the original Wendt–Abraham criterion we obtain a significantly overestimated *T_g_*. We also emphasise that the decrease of the coordination number with the increase of temperature alone cannot explain the glass transition because such changes can be caused by rearrangements of crystalline lattice and the change of FSDM is the crucial parameter to be checked for eventual changes signalling on phase transformations in amorphous materials. 

## 6. Conclusions

In conclusion we have used the connection between atomic shifts which can be detected via X-ray diffraction and formation of configurons which are broken chemical bonds in amorphous materials. We have analysed the temperature behaviour of FSDM (minima of *PDF*(*r*) as a function of temperature), identifying the temperature where the dependence exhibits a kink and allocating that temperature to the glass transition temperature, *T_g_* = 794 ± 10 K at the cooling rate of 1 × 10^13^ K/s. It has been found that for amorphous Cu the method based on analysis of FSDM is more sensitive compared with the empirical criterion of Wendt−Abraham. 

## Figures and Tables

**Table 1:** The parameters of linear dependences of *PDF_min_*(*T*) and *PDF_min_*(*T*)*/PDF_max_*(*T*).

a_g_	b_g_	c_g_	d_g_	a_l_	b_l_	c_l_	d_g_
0.0001635	0.2080342	0.0000757	0.0394081	0.0002568	0.1339328	0.0001478	−0.0163428

## Data Availability

Data supporting the research are within the paper.
